# Phenotypic characteristics of F64L, I68L, I107V, and S77Y ATTRv genotypes from the Transthyretin Amyloidosis Outcomes Survey (THAOS)

**DOI:** 10.1371/journal.pone.0292435

**Published:** 2024-01-19

**Authors:** Luca Gentile, Igor Diemberger, Violaine Plante-Bordeneuve, Anna Mazzeo, Amir Dori, Marco Luigetti, Andrea Di Paolantonio, Angela Dispenzieri, Martha Grogan, Márcia Waddington Cruz, David Adams, Jocelyn Inamo, Arnt V. Kristen, Calogero Lino Cirami, Doug Chapman, Pritam Gupta, Oliver Glass, Leslie Amass

**Affiliations:** 1 Department of Clinical and Experimental Medicine, Università of Messina, Messina, Italy; 2 University of Bologna, Department of Medical and Surgical Sciences, Bologna, Italy; 3 Cardiology Unit, IRCCS Azienda Ospedaliero-Universitaria di Bologna, Bologna, Italy; 4 Hopital Henri Mondor, Assistance Publique‐Hopitaux de Paris, East Paris‐Créteil University, Créteil, France; 5 Department of Neurology, Sheba Medical Center, Ramat Gan, and Tel-Aviv University, Tel-Aviv, Israel; 6 Fondazione Policlinico Universitario A. Gemelli IRCCS, Rome, Italy; 7 Universita Cattolica del Sacro Cuore, Rome, Italy; 8 U.O. Neurologia, Fondazione Poliambulanza, Brescia, Italy; 9 Division of Hematology, Mayo Clinic, Rochester, Minnesota, United States of America; 10 Department of Cardiovascular Diseases, Mayo Clinic, Rochester, Minnesota, United States of America; 11 National Amyloidosis Referral Center, CEPARM, Federal University of Rio de Janeiro, Rio de Janeiro, Brazil; 12 Department of Neurology, CHU de Bicêtre, AP-HP, University Paris-Saclay, Le Kremlin-Bicêtre, France; 13 CHU de Fort de France, Fort de France, Martinique, France; 14 Department of Cardiology, Angiology, and Respiratory Medicine, Medical University of Heidelberg, Heidelberg, Germany; 15 Nephrology Department, Azienda Ospedaliero-Universitaria Careggi, Florence, Italy; 16 Pfizer Inc, New York, New York, United States of America; Consejo Superior de Investigaciones Cientificas, SPAIN

## Abstract

Transthyretin amyloidosis (ATTR amyloidosis) is a progressive, multi-systemic disease with wild-type (ATTRwt) and hereditary (ATTRv) forms. Over 130 variants associated with ATTRv amyloidosis have been identified, although little is known about the majority of these genotypes. This analysis examined phenotypic characteristics of symptomatic patients with ATTRv amyloidosis enrolled in the Transthyretin Amyloidosis Outcomes Survey (THAOS) with four less frequently reported pathogenic genotypes: F64L (c.250T>C, p.F84L), I68L (c.262A>T, p.I88L), I107V (c.379A>G; p.I127V), and S77Y (c.290C>A; p.S97Y). THAOS is the largest ongoing, global, longitudinal observational study of patients with ATTR amyloidosis, including both ATTRwt and ATTRv amyloidosis. This analysis describes the baseline demographic and clinical characteristics of untreated symptomatic patients with the F64L, I68L, I107V, or S77Y genotypes at enrollment in THAOS (data cutoff date: January 4, 2022). There were 141 symptomatic patients with F64L (n = 46), I68L (n = 45), I107V (n = 21), or S77Y (n = 29) variants at the data cutoff. Most patients were male and median age at enrollment was in the sixth decade for S77Y patients and the seventh decade for the others. A predominantly neurologic phenotype was associated with F64L, I107V, and S77Y genotypes, whereas patients with the I68L genotype presented with more pronounced cardiac involvement. However, a mixed phenotype was also reported in a considerable proportion of patients in each variant subgroup. This analysis from THAOS represents the largest study of ATTRv symptomatic patients with the F64L, I68L, I107V, and S77Y genotypes. These data add to the limited knowledge on the clinical profile of patients with specific ATTRv variants and emphasize the importance of comprehensive assessment of all patients.

Trial registration

ClinicalTrials.gov: NCT00628745.

## Introduction

Transthyretin amyloidosis (ATTR amyloidosis) is a progressive, multi-systemic disease with wild-type (ATTRwt) and hereditary (ATTRv) forms [1]. ATTRv amyloidosis is caused by variants in the transthyretin (*TTR*) gene that destabilize the TTR protein, leading to systemic deposition of TTR amyloid fibrils and impairment mainly to the peripheral nerves, autonomic nervous system, and heart [2, 3]. The phenotypic presentation of ATTRv amyloidosis is clinically heterogeneous, with phenotypes ranging from predominantly neurologic to predominantly cardiac to mixed, depending on the particular *TTR* variant and other factors such as age of onset, disease penetrance, and geographic location [4–7]. If untreated, patients’ survival estimates range from 2 to 10 years [1, 4]. Over 130 different *TTR* variants associated with ATTRv amyloidosis have been identified [8]. V30M (c.148G>A; p.V50M) is the most frequently reported *TTR* variant, representing approximately 70% of patients worldwide [9], whereas many other genotypes are not well characterized.

Although limited data are available, there have been prior descriptions of these variants. The F64L (c.250T>C, p.F84L) variant is responsible for a late-onset, predominantly neurologic form of ATTRv amyloidosis [10, 11] characterized by a high number of sporadic patients, high male/female ratio, and carpal tunnel syndrome (CTS) frequently reported at onset [12]. The I68L (c.262A>T, p.I88L) variant causes a late-onset, predominantly cardiac form of ATTRv amyloidosis, mimicking a hypertrophic cardiomyopathy that is almost indistinguishable from ATTRwt amyloidosis [13, 14]. The I107V (c.379A>G, p.I127V) variant has been most frequently described in France and Japan, with clinical presentations of CTS, polyneuropathy, late-onset cardiomyopathy, and possible cranial nerve involvement [15–17]. The S77Y (c.290C>A, p.S97Y) variant is particularly frequent in northern France, with a predominantly neurologic phenotype and demyelinating pattern reported by nerve conduction studies (NCS) [18, 19].

The Transthyretin Amyloidosis Outcomes Survey (THAOS) is an ongoing, global, longitudinal, observational survey of patients with ATTR amyloidosis, including both hereditary and wild-type disease, and asymptomatic carriers with TTR variants [20]. THAOS collects data on the natural history of the disease from a large and diverse patient population to better characterize forms of ATTR amyloidosis and improve disease diagnosis and patient management. This analysis of THAOS data was conducted to gain a deeper understanding of the characteristics of patients with the F64L, I68L, I107V, or S77Y variant.

## Methods

The design and methodology of THAOS (ClinicalTrials.gov: NCT00628745) have been previously described in detail [20]. This analysis describes the baseline characteristics of untreated symptomatic patients with the F64L, I68L, I107V, or S77Y variant at enrollment in THAOS starting from its onset in 2007 to the data cutoff date of January 4, 2022. Authors did not have access to identifying information after data collection. Symptomatic patients were defined as patients with at least one symptom rated by investigators as definitely related to ATTR amyloidosis at enrollment who were not treated with tafamidis before or on the date of enrollment. Symptom duration was defined as the time from the onset of symptom(s) considered definitely related to ATTR amyloidosis to enrollment. Neurologic impairment was assessed using the Neuropathy Impairment Score in the Lower Limbs (NIS-LL; range 0–88), with higher scores indicating greater impairment [21], and with the modified polyneuropathy disability (mPND) score (range I–IV), where I indicates sensory disturbance in lower limbs but preserved walking capacity; II indicates difficulties in walking but no need of a walking stick; IIIa indicates one stick or one crutch required for walking; IIIb indicates two sticks or two crutches required for walking; and IV indicates patient confined to a wheelchair or bed. Quality of life was assessed using the Karnofsky Performance Status score (range 0–100%), and EQ-5D-3L index score (ranges from below 0 to 1) [22], with lower scores indicating greater impairment. Nutritional status was evaluated using the body mass index (BMI) and the modified BMI (mBMI), calculated by multiplying the BMI by serum albumin level to compensate for fluid accumulation.

Patients were classified by phenotype at enrollment based on the following definitions:

- **Predominantly cardiac phenotype:**
   ⚬ abnormal electrocardiogram (ECG) due to rhythm disturbance or heart failure or dyspnea;
   ⚬ no more than mild neurologic or gastrointestinal (GI) symptoms (excluding erectile dysfunction, constipation, and carpal tunnel).

Cardiac symptoms did not need to be ongoing at a given visit to be included for phenotyping; however, symptoms had to be definitely related to ATTR amyloidosis as judged by the investigator.

- **Predominantly neurologic phenotype:**
   ⚬ neurologic symptoms of any severity or GI symptoms of any severity;
   ⚬ no abnormal ECG due to rhythm disturbance or heart failure or dyspnea.

Neurologic and GI symptoms had to be ongoing and definitely related to ATTR amyloidosis. An mPND score ≥I was included as a neurologic symptom wherever applicable.

- **Mixed phenotype:**
   ⚬ abnormal ECG due to rhythm disturbance or heart failure or dyspnea;
   ⚬ neurologic symptoms of any severity or GI symptoms of any severity but did not satisfy the criteria for predominantly cardiac or predominantly neurologic.
- **Symptomatic patients with unknown phenotype:**
   ⚬ all other symptomatic patients who did not meet any of the above criteria for either predominantly cardiac, predominantly neurologic, or mixed phenotypes.

All THAOS study sites received ethical or institutional review board approval prior to patient enrollment, and each patient provided written informed consent. The study followed the International Council for Harmonisation Good Pharmacoepidemiology Practice guidelines and the principles of the Declaration of Helsinki.

Continuous data are presented as mean (standard deviation [SD]) or median (10th, 90th percentile), and categorical data are presented as count (percentage). SAS software (SAS Institute; Cary, North Carolina; version 9.4) was used for data analysis.

## Results

### F64L

**Fig 1:**
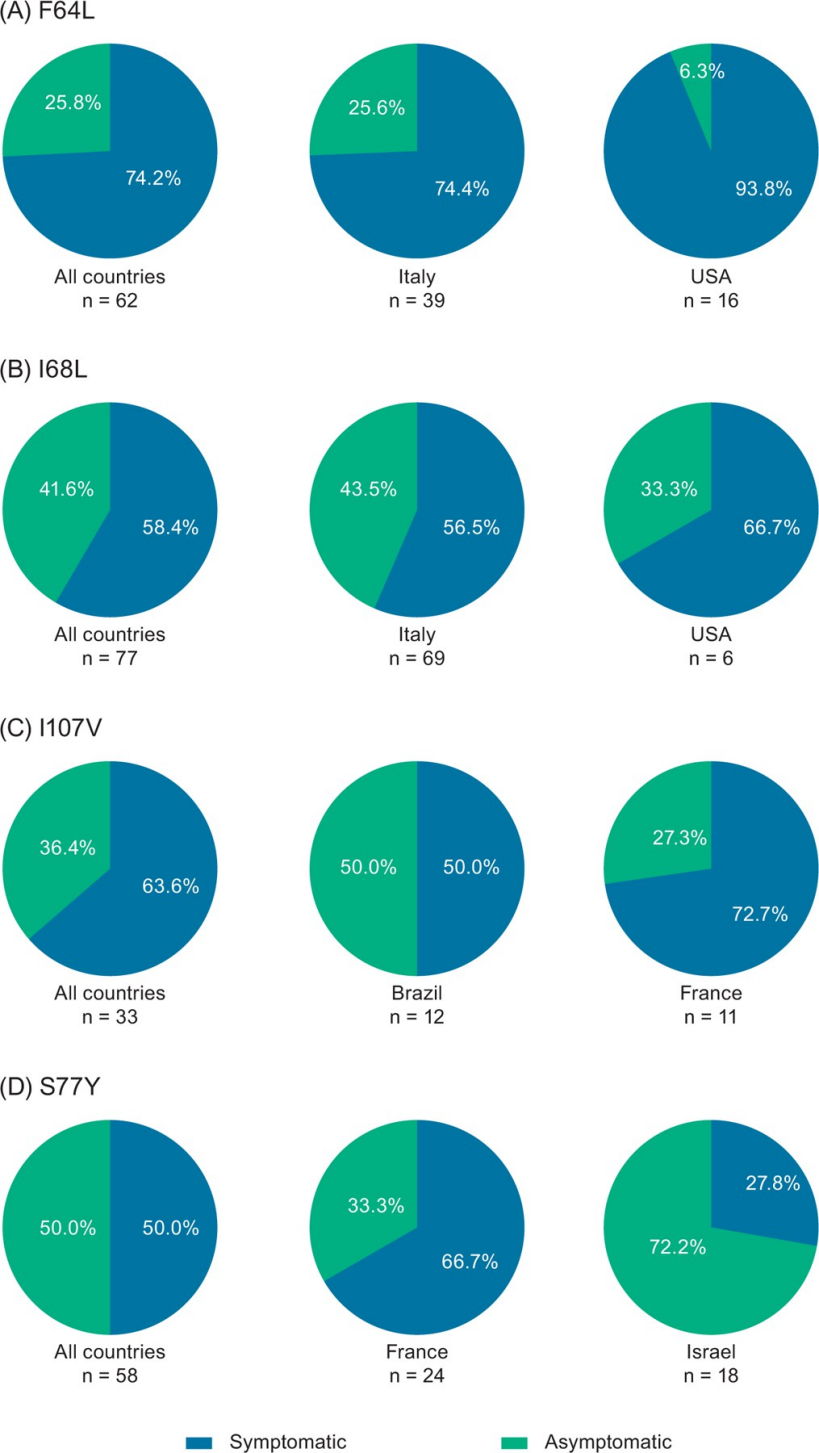
Symptom status of all patients with ATTRv amyloidosis and the (A) F64L, (B) I68L, (C) I107V, and (D) S77Y variants in THAOS. Symptom status is shown across all countries and in countries with the largest number of patients with the given variant. ATTRv amyloidosis, hereditary transthyretin amyloidosis; THAOS, Transthyretin Amyloidosis Outcomes Survey.

**Fig 2:**
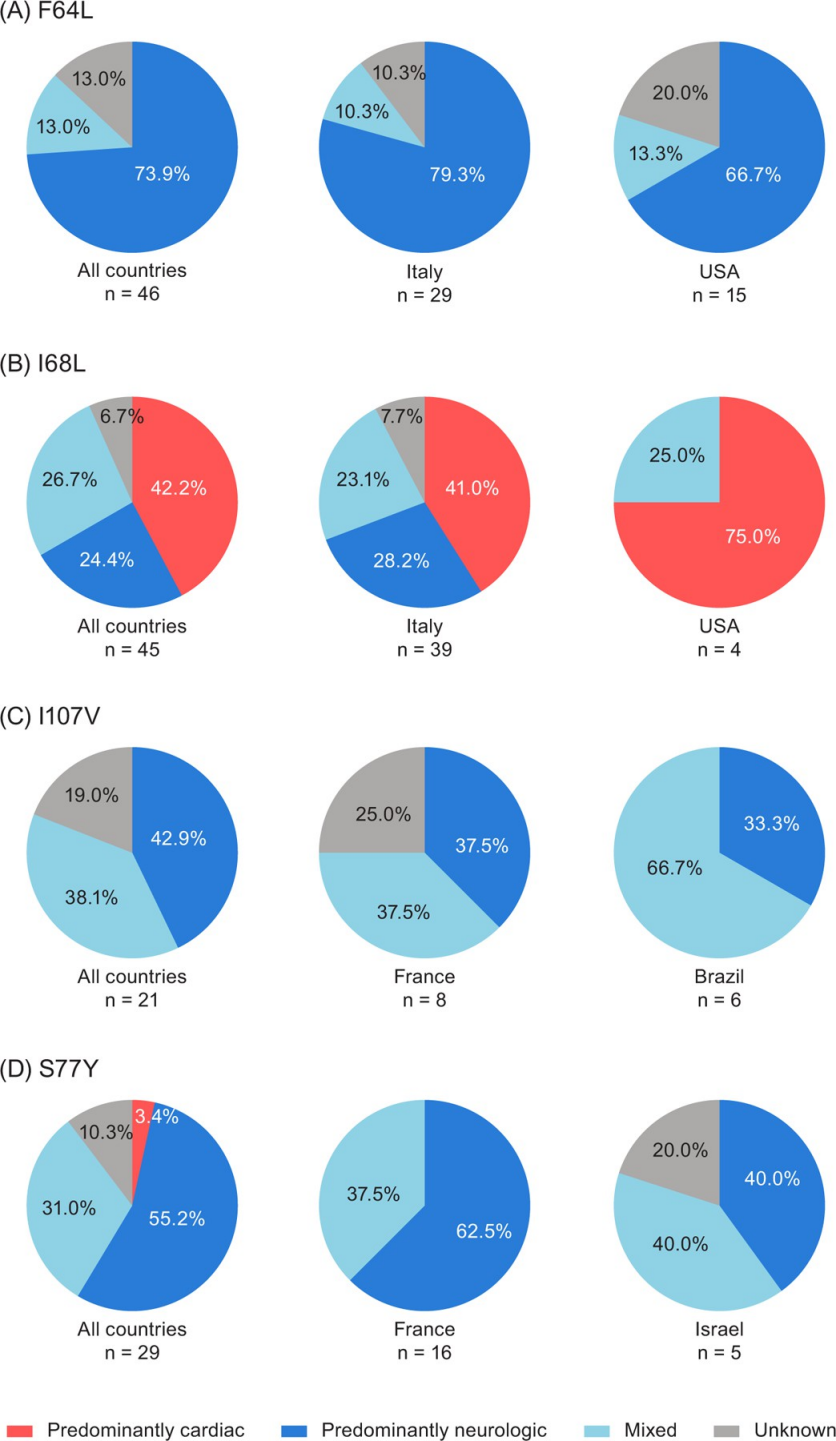
Phenotype distribution in symptomatic patients with ATTRv amyloidosis and the (A) F64L, (B) I68L, (C) I107V, and (D) S77Y variants in THAOS. Phenotype distribution is shown across all countries and in countries with the largest number of patients with the given variant. ATTRv amyloidosis, hereditary transthyretin amyloidosis; THAOS, Transthyretin Amyloidosis Outcomes Survey.

**Fig 3:**
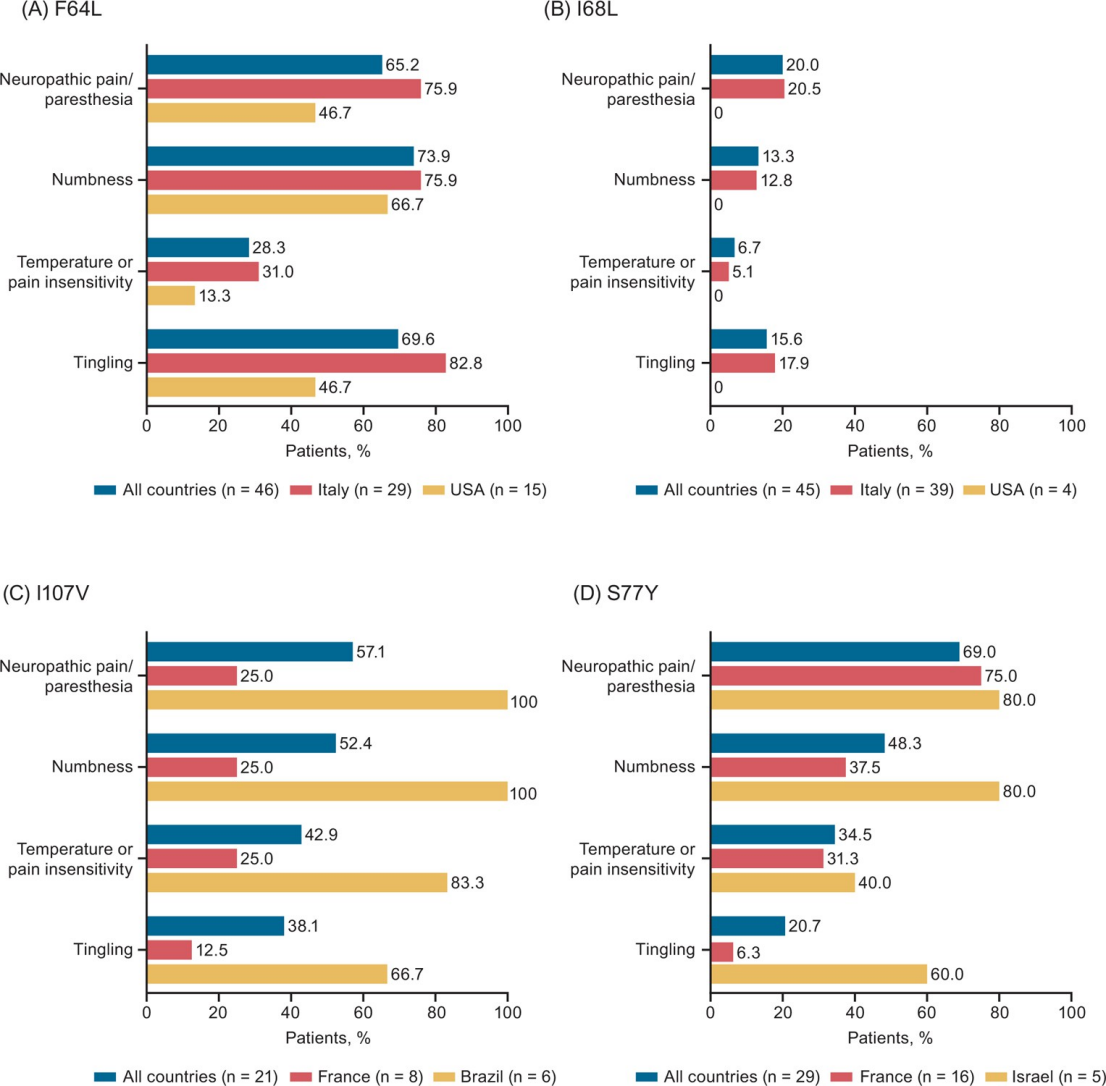
Neurologic symptoms in symptomatic patients with ATTRv amyloidosis and the (A) F64L, (B) I68L, (C) I107V, and (D) S77Y variants in THAOS. Neurologic symptoms are shown across all countries and in countries with the largest number of patients with the given variant. ATTRv amyloidosis, hereditary transthyretin amyloidosis; THAOS, Transthyretin Amyloidosis Outcomes Survey.

**Fig 4:**
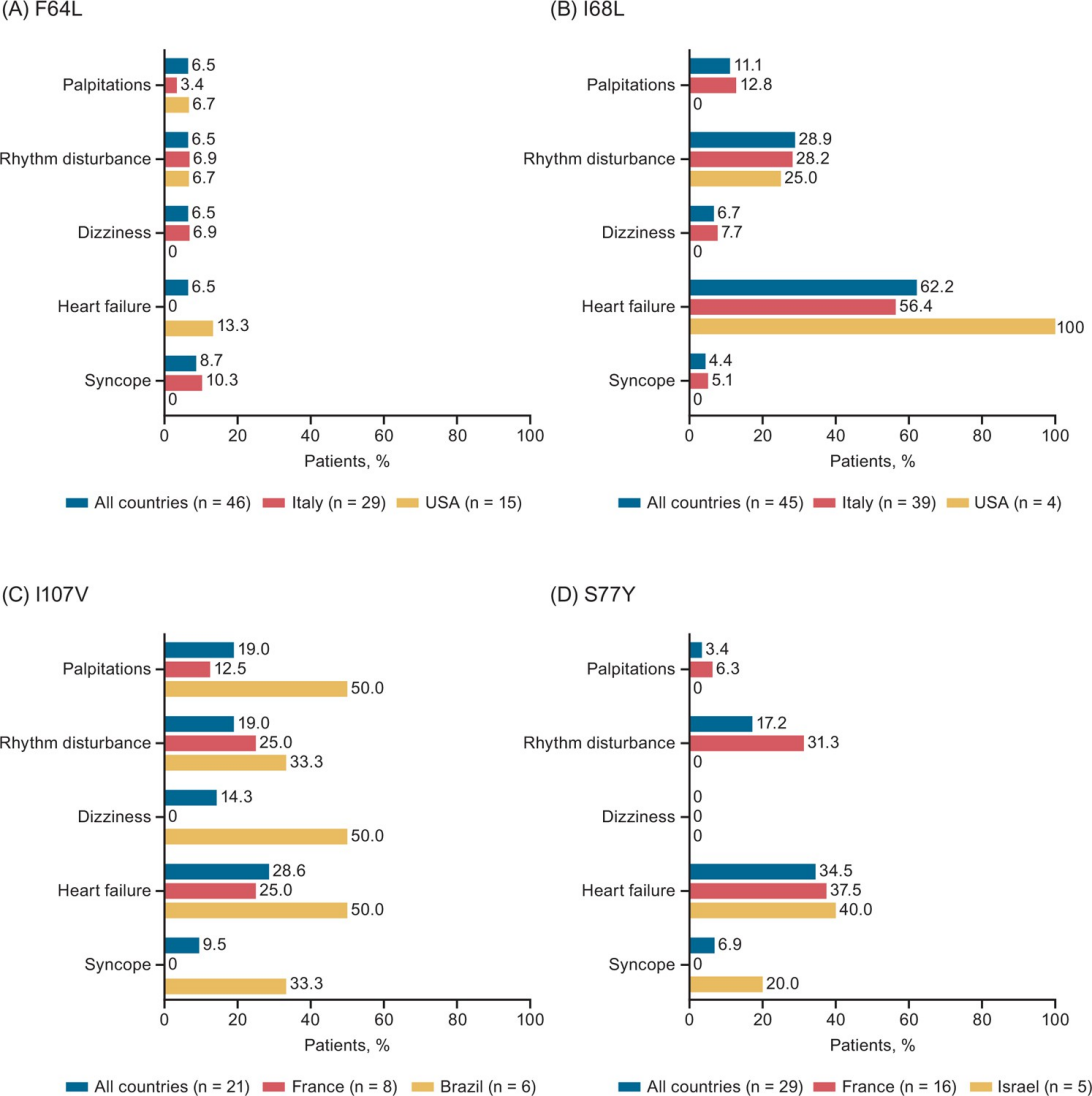
Cardiac manifestations in symptomatic patients with ATTRv amyloidosis and the (A) F64L, (B) I68L, (C) I107V, and (D) S77Y variants in THAOS. Cardiac manifestations are shown across all countries and in countries with the largest number of patients with the given variant. ATTRv amyloidosis, hereditary transthyretin amyloidosis; THAOS, Transthyretin Amyloidosis Outcomes Survey.

A total of 62 patients had the F64L variant in THAOS; 46 (74.2%) of these patients were symptomatic at enrollment (Fig 1). Of 46 symptomatic patients (31 males, 67.4%), 29 (63.0%) were enrolled in Italy, 15 (32.6%) in the United States, and the remaining 2 (4.4%) in Brazil and Argentina (S1 Table). Among symptomatic patients, median age at enrollment was 67.6 years, with a median duration of symptoms of 5.0 years (Table 1). Most symptomatic patients (73.9%) had a predominantly neurologic phenotype (Fig 2), with a median NIS-LL derived total score of 23.6 (Table 1). Of 40 symptomatic patients with a predominantly neurologic or mixed phenotype at enrollment, 40% of patients had an mPND score ≥2 (Table 2). The predominantly neurologic phenotype was common in Italy (79.3%). None of the symptomatic F64L patients had a predominantly cardiac phenotype. Median mBMI was 1083.5, and median EQ-5D-3L index score was 0.60. More than half of the patients (53.0%) were able to care for themselves (Karnofsky index 70–90). Of six symptomatic patients with a mixed phenotype at enrollment, 50% had conduction abnormalities at baseline, including first degree atrioventricular block (n = 2) and left anterior hemiblock (n = 1). The left ventricular (LV) diastolic diameter was collected for only one patient and it was 36.2 mm (Table 3). Overall, numbness was the most common neurologic symptom, followed by tingling and neuropathic pain/paresthesia; syncope was the most common cardiac manifestation (Figs 3 and 4).

**Table 1:** Baseline demographic and clinical characteristics of symptomatic patients with ATTRv amyloidosis and the F64L, I68L, I107V, or S77Y variant in THAOS.

Characteristic	F64L (n = 46)	I68L (n = 45)	I107V (n = 21)	S77Y (n = 29)
Male	31 (67.4)	35 (77.8)	18 (85.7)	21 (72.4)
Age at enrollment (years)	67.6 (52.1, 76.4)	69.3 (56.5, 80.4)	63.7 (56.7, 76.0)	57.8 (47.2, 69.6)
BMI	23.8 (19.1, 32.3)	25.6 (22.0, 32.4)	25.9 (19.5, 32.6)	25.3 (19.9, 33.1)
mBMI	1083.5 (805.2, 1389.1)	1106.4 (690.9, 1539.8)	879.1 (824.9, 1446.2)	978.0 (564.0, 1228.6)
Symptom duration (years)	5.0 (0.8, 13.4)	3.0 (0.7, 11.9)	3.6 (1.2, 10.6)	4.7 (1.2, 12.7)
EQ-5D-3L index score	0.6 (0.31, 0.82)	0.8 (0.6, 1.0)	0.6 (0.1, 0.9)	0.7 (0.7, 0.8)
Derived NIS-LL total score^a^	23.6 (2.0, 61.0)	2.0 (0.0, 20.0)	38.9 (2.0, 65.9)	8.0 (0.0, 72.5)
Karnofsky Performance Status score^b^				
10–30	0	0	0	1 (4.8)
40–60	10 (29.5)	4 (9.7)	7 (41.2)	4 (19.1)
70–90	18 (53.0)	28 (68.3)	7 (41.2)	12 (57.1)
100	6 (17.6)	9 (22.0)	3 (17.6)	4 (19.0)

Values are n (%) or median (10^th^, 90^th^ percentile). In the respective groups (F64L, I68L, I107V, and S77Y), BMI was available in 46, 45, 18, and 27 patients; mBMI in 22, 9, 10 and 10 patients; EQ-5D-3L in 20, 39, 12, and 2 patients; and Derived NIS-LL total score in 12, 4, 10, and 7 patients.

^a^NIS-LL score was derived by an algorithm using symptoms.

^b^Percentages based on number of patients with available scores.

ATTRv amyloidosis, hereditary transthyretin amyloidosis; BMI, body mass index; mBMI, modified body mass index; NIS-LL, Neuropathy Impairment Score in the Lower Limbs; THAOS, Transthyretin Amyloidosis Outcomes Survey.

**Table 2:** mPND score distribution of symptomatic patients with a predominantly neurologic or mixed phenotype at enrollment with ATTRv amyloidosis and the F64L, I68L, I107V, or S77Y variant in THAOS.

Characteristic	F64L (n = 40)	I68L (n = 23)	I107V (n = 17)	S77Y (n = 25)
mPND score, n (%)				
0	3 (7.5)	4 (17.4)	1 (5.9)	2 (8)
I	11 (27.5)	12 (52.2)	5 (29.4)	11 (44)
II	6 (15)	3 (13)	3 (17.6)	2 (8)
IIIa	4 (10)	2 (8.7)	0	1 (4)
IIIb	3 (7.5)	1 (4.3)	5 (29.4)	2 (8)
IV	3 (7.5)	0	2 (11.8)	3 (12)
Missing	10 (25)	1 (4.3)	1 (5.9)	4 (16)

ATTRv amyloidosis, hereditary transthyretin amyloidosis; mPND, modified polyneuropathy disability; THAOS, Transthyretin Amyloidosis Outcomes Survey.

**Table 3:** Cardiac measures in symptomatic patients with a predominantly cardiac or mixed phenotype at enrollment with ATTRv amyloidosis and the F64L, I68L, I107V, or S77Y variant in THAOS.

Characteristic	F64L (n = 6)	I68L (n = 31)	I107V (n = 8)	S77Y (n = 10)
Atrial fibrillation or atrial flutter				
Yes	0	9 (29.0)	0	3 (30.0)
No	1 (16.7)	2 (6.5)	1 (12.5)	0
Missing	5 (83.3)	20 (64.5)	7 (87.5)	7 (70.0)
Conduction abnormalities				
Yes	3 (50.0)	18 (58.1)	4 (50.0)	4 (40.0)
No	0	5 (16.1)	2 (25.0)	2 (20.0)
Missing	3 (50.0)	8 (25.8)	2 (25.0)	4 (40.0)
Pacemaker				
Yes	1 (16.7)	3 (9.7)	1 (12.5)	4 (40.0)
No	5 (83.3)	11 (35.5)	2 (25.0)	0
Missing	0	17 (54.8)	5 (62.5)	6 (60.0)
LV diastolic diameter (mm)	36.2 (36.2, 36.2)	46.0 (43.0, 54.0)	42.0 (40.0, 48.0)	52.0 (33.0, 55.0)
LV systolic diameter (mm)	20.3 (20.3, 20.3)	36.0 (25.0, 44.0)	31.5 (27.0, 35.0)	43.0 (29.0, 45.0)
LV septum diameter (mm)	16.0 (16.0, 16.0)	17.0 (14.0, 21.0)	18.0 (13.0, 23.0)	21.0 (11.0, 22.0)
Ejection fraction (%)	73.0 (73.0, 73.0)	52.0 (35.0, 62.0)	53.0 (45.0, 66.0)	51.5 (38.0, 66.0)
LV mass index (gm/m^2^)	90.8 (90.8, 90.8)	186.4 (140.2, 248.3)	171.5 (121.9, 179.4)	159.0 (104.4, 297.6)
NT-proBNP (pg/mL)	527.9 (208.0, 847.7)	2648.0 (1149.0, 3782.0)	1009.0 (803.0, 1215.0)	3555.5 (325.0, 7406.8)

Values are n (%) or median (10^th^, 90^th^ percentile). In the respective groups (F64L, I68L, I107V, S77Y), LV diastolic diameter was available in 1, 13, 5 and 5 patients; LV systolic diameter in 1, 13, 4, and 4 patients; LV septum diameter in 1, 18, 5, and 5 patients; Ejection fraction in 1, 19, 5, and 4 patients; LV mass index in 1, 13, 4, and 4, patients; and NT-proBNP in 2, 11, 2, and 4 patients.

ATTRv amyloidosis, hereditary transthyretin amyloidosis; LV, left ventricular; NT-proBNP, N-terminal pro-B-type natriuretic peptide.

### I68L

There were 77 patients (68.8% male) with the I68L variant in THAOS, 45 (58.4%) of whom were symptomatic at enrollment (Fig 1). Of 45 symptomatic patients (35 males, 77.8%), 39 (86.7%) were enrolled in Italy, 4 (8.9%) in the United States, and 1 (2.2%) each in Germany and France (S2 Table). Median age at enrollment was 69.3 years, and median symptom duration was 3.0 years (Table 1). Predominantly cardiac was the most common phenotype, followed by mixed and predominantly neurologic (Fig 2). More than 50% of the symptomatic patients who were predominantly neurologic or mixed at enrollment had an mPND score of I (Table 2). Median mBMI was 1106.4, and median EQ-5D-3L index score was 0.83. Most patients (68.3%) were able to care for themselves (Karnofsky index 70–90) (Table 1). Of 31 symptomatic patients with a predominantly cardiac or mixed phenotype at enrollment, the following were observed: 9 (29%) had atrial fibrillation or atrial flutter at baseline, 18 (58.1%) had conduction abnormalities, 3 (9.7%) had a pacemaker, median LV diastolic diameter was 46 mm, median LV septum diameter was 17 mm, median ejection fraction was 52%, median LV mass index was 186.4 gm/m^2^, and median N-terminal pro-B-type natriuretic peptide (NT-proBNP) was 2648 pg/mL (Table 3). Heart failure was the most common cardiac manifestation (62.2% of patients), followed by rhythm disturbance (28.9%), whereas neuropathic pain/paresthesia was the most common neurologic symptom (Figs 3 and 4).

### I107V

A total of 33 patients (66.7% males) with the I107V variant were in THAOS, 21 (63.6%) of whom were symptomatic at enrollment (Fig 1). Of the 21 symptomatic patients (18 males, 85.7%), 8 (38.1%) were enrolled in France, 6 (28.6%) in Brazil, and the remaining 7 (33.3%) in Germany, Japan, and the United States (S3 Table). Median age at enrollment was 63.7 years, with a median symptom duration of 3.6 years (Table 1). Predominantly neurologic was the most common phenotype (42.9%), with a median NIS-LL of 38.9; almost 59% of symptomatic patients with a predominantly neurologic or mixed phenotype at enrollment had an mPND score ≥2 (Table 2). In France, a predominantly neurologic and mixed phenotype (both 37.5%) were equally as common, whereas the most common phenotype in Brazil was mixed (66.7%) (Fig 2). Overall, median mBMI was 879.1, and median EQ-5D-3L index score was 0.64. Almost 41% of patients were able to care for themselves (Karnofsky index 70–90), while a similar percentage (41.2%) had greater disability (Karnofsky index 40–60) (Table 1). Of eight symptomatic patients with a predominantly cardiac or mixed phenotype at enrollment, 50% had conduction abnormalities, median LV diastolic diameter was 42 mm, median LV septum diameter was 18 mm, median ejection fraction was 53%, and median LV mass index was 171.5 gm/m^2^ (Table 3). Overall, heart failure was the most common cardiac manifestation, reported by 28.6% of patients, and over half reported neuropathic pain/paresthesia (57.1%) and/or numbness (52.4%) (Figs 3 and 4).

### S77Y

There were 58 patients (67.2% male) with the S77Y variant in THAOS, 29 (50.0%) of whom were symptomatic at enrollment. Of the 29 symptomatic patients (21 males, 72.4%), most were enrolled in France (n = 16 [55.2%]), Israel (n = 5 [17.2%]), or the United States (n = 5 [17.2%]) (S4 Table). Median age at enrollment was 57.8 years, with a median symptom duration of 4.7 years (Table 1). The most common phenotype was predominantly neurologic (55.2%) (Fig 2), with a median NIS-LL derived total score of 8; 44% of symptomatic patients with a predominantly neurologic or mixed phenotype at enrollment had an mPND score of I (Table 2). Median mBMI was 978.0, and median EQ-5D-3L index score was 0.74. About 57% were able to care for themselves (Karnofsky index 70–90) (Table 1). Out of 10 symptomatic patients with a predominantly cardiac or mixed phenotype at enrollment, 40% had a pacemaker. The median LV diastolic diameter was 52 mm and median LV mass index was 150 gm/m^2^ (Table 3). The most common cardiac manifestation overall was heart failure, and the most common neurologic symptom was neuropathic pain/paresthesia (69.0% of patients) (Figs 3 and 4).

## Discussion

Over 130 variants in the *TTR* gene have been identified to cause pathogenic ATTRv amyloidosis, with very few (V30M, V122I [c.424G>A; p.V142I]) being sufficiently described in the literature. With the wealth of data available in THAOS [23], there is an opportunity to gain knowledge and describe these less prominent variants. Even in THAOS, among all symptomatic patients enrolled, 75% are affected by ATTRwt amyloidosis or by ATTRv amyloidosis caused by V30M or V122I variants [23]. The remaining 25% patients have less frequently recognized variants, usually described in the literature by relatively short case series or case reports. The current analysis provides the largest evaluation of the clinical characteristics of ATTRv symptomatic patients with the F64L, I68L, I107V, or S77Y genotypes to date.

Age at enrollment was comparable between F64L, I68L, and I107V patients, while S77Y subjects were younger at enrollment, and with the longest symptom duration, particularly patients from France with the S77Y variant. Geographical distribution in THAOS is consistent with previous reports, with F64L and I68L variants frequently identified in Italy, S77Y in France and Israel, and I107V in Brazil [9–15, 17–19, 24]. Male predominance was confirmed across all genotypes, most notably in patients with the I107V variant [9–12, 14, 19, 23, 24]. Mitochondrial DNA defects and neurohormonal factors have been taken into consideration as possible causes of these gender-related differences [25, 26], but further studies are needed to confirm the role of these factors.

A predominantly neurologic phenotype was associated with F64L, I107V, and S77Y variants. In particular, 79.3% of Italian F64L patients presented with almost exclusively neuropathic symptoms, compared with 66.7% of patients from the United States with the same variant. The I68L variant was the only variant with the greatest proportion of patients having a predominantly cardiologic phenotype (42.2%). Another difference was the mPND score distribution of symptomatic patients with a predominantly neurologic or mixed phenotype at enrollment, with 17.4% of I68L patients in class 0 (no neurologic symptoms), while a large number of F64L, I107V, and S77Y patients were in class 2 or more (40%, 58.8%, and 32.0%, respectively). Specifically, 41.2% of symptomatic I107V patients and 25.0% of symptomatic F64L subjects with a predominantly neurologic or mixed phenotype at enrollment were not able to walk independently (mPND score ≥IIIa), while S77Y patients had milder neuropathic limitations (52.2% with an mPND score of I). However, a mixed phenotype was also reported in a considerable proportion of patients for each (13–38% of patients), with some interesting regional differences. In fact, Brazilian I107V patients presented with a mixed phenotype two times more frequently than Italian and French patients, who mainly had classic neurologic involvement. Finally, I68L variants were, as expected, mainly associated with a predominantly cardiac phenotype (42.2% of symptomatic subjects). In these patients, conduction abnormalities were the most common signs of cardiomyopathy at ECG, while echocardiogram revealed an increase in LV diastolic diameter and LV septum diameter. As reported by previous studies, normal median ejection fraction (52%) was observed, despite laboratory evidence of cardiac dysfunction (median NT-proBNP: 2648 pg/mL). Although there is a prevalent cardiac manifestation of ATTRv disease in this variant, a considerable number of symptomatic patients presented with a mixed (26.7%) or predominantly neurologic (24.4%) phenotype associated with a milder neurologic impairment (mPND score 1 in more than 50% of these patients). These data confirm that patients with ATTRv amyloidosis must be thoroughly investigated, even if harboring variants historically associated with a neurologic or cardiologic phenotype.

THAOS has notable strengths in that the registry represents a large, geographically diverse group of countries and study sites worldwide. Study limitations include the overall smaller number of patients available to analyze, and referral bias may have impacted phenotype categorization which has the potential to change the clinical and epidemiologic aspects of the variants evaluated here. Registry data, by nature, are also not always complete and are limited by the data imputed. Lastly, the current analysis presents baseline data only; future studies should examine prognostic data to provide insight into long-term outcomes of these patients.

## Conclusions

ATTRv amyloidosis is a heterogeneous genetic disease, with the V30M genotype being the most common variant reported and described worldwide. Other genotypes are less frequent, and the corresponding phenotypes are not well characterized. THAOS continues to accumulate valuable real-world data from patients with ATTR amyloidosis, including those with less frequently reported pathogenic genotypes, such as F64L, I68L, I107V, and S77Y. The findings presented in this analysis enhance the current understanding of the clinical profile of patients with these genotypes and emphasize the importance of comprehensive assessment of all patients.

## Supporting information

S1 Table Baseline demographic and clinical characteristics of symptomatic patients with ATTRv amyloidosis and the F64L variant in THAOS, detailed by country of origins. (DOCX)

S2 Table Baseline demographic and clinical characteristics of symptomatic patients with ATTRv amyloidosis and the I68L variant in THAOS, detailed by country of origins. (DOCX)

S3 Table Baseline demographic and clinical characteristics of symptomatic patients with ATTRv amyloidosis and the I107V variant in THAOS, detailed by country of origins. (DOCX)

S4 Table Baseline demographic and clinical characteristics of symptomatic patients with ATTRv amyloidosis and the S77Y variant in THAOS, detailed by country of origins. (DOCX)

S1 Checklist STROBE statement—checklist of items that should be included in reports of observational studies. (DOCX)

## References

[1] Ruberg FL, Grogan M, Hanna M, Kelly JW, Maurer MS. Transthyretin amyloid cardiomyopathy: JACC state-of-the-art review. J Am Coll Cardiol. 2019;73(22):2872–91. Epub 2019/06/07. doi: 10.1016/j.jacc.2019.04.003 ; PubMed Central PMCID: PMC6724183. 31171094 PMC6724183

[2] Plante-Bordeneuve V, Said G. Familial amyloid polyneuropathy. Lancet Neurol. 2011;10(12):1086–97. Epub 2011/11/19. doi: 10.1016/S1474-4422(11)70246-0 . 22094129

[3] Adams D, Koike H, Slama M, Coelho T. Hereditary transthyretin amyloidosis: a model of medical progress for a fatal disease. Nat Rev Neurol. 2019;15(7):387–404. Epub 2019/06/19. doi: 10.1038/s41582-019-0210-4 . 31209302

[4] Ando Y, Coelho T, Berk JL, Cruz MW, Ericzon BG, Ikeda S, et al. Guideline of transthyretin-related hereditary amyloidosis for clinicians. Orphanet J Rare Dis. 2013;8:31. Epub 2013/02/22. doi: 10.1186/1750-1172-8-31 ; PubMed Central PMCID: PMC3584981. 23425518 PMC3584981

[5] Adams D, Ando Y, Beirao JM, Coelho T, Gertz MA, Gillmore JD, et al. Expert consensus recommendations to improve diagnosis of ATTR amyloidosis with polyneuropathy. J Neurol. 2021;268(6):2109–22. Epub 2020/01/08. doi: 10.1007/s00415-019-09688-0 ; PubMed Central PMCID: PMC8179912. 31907599 PMC8179912

[6] Ruberg FL, Berk JL. Transthyretin (TTR) cardiac amyloidosis. Circulation. 2012;126(10):1286–300. Epub 2012/09/06. doi: 10.1161/CIRCULATIONAHA.111.078915 ; PubMed Central PMCID: PMC3501197. 22949539 PMC3501197

[7] Yamamoto H, Yokochi T. Transthyretin cardiac amyloidosis: an update on diagnosis and treatment. ESC Heart Fail. 2019;6(6):1128–39. Epub 2019/09/26. doi: 10.1002/ehf2.12518 ; PubMed Central PMCID: PMC6989279. 31553132 PMC6989279

[8] Rowczenio D, Wechalekar A. Mutations in Hereditary Amyloidosis 2015 [October 11, 2021]. Available from: http://amyloidosismutations.com/mut-attr.php.

[9] Russo M, Obici L, Bartolomei I, Cappelli F, Luigetti M, Fenu S, et al. ATTRv amyloidosis Italian Registry: clinical and epidemiological data. Amyloid. 2020;27(4):259–65. Epub 2020/07/23. doi: 10.1080/13506129.2020.1794807 . 32696671

[10] Russo M, Mazzeo A, Stancanelli C, Di Leo R, Gentile L, Di Bella G, et al. Transthyretin-related familial amyloidotic polyneuropathy: description of a cohort of patients with Leu64 mutation and late onset. J Peripher Nerv Syst. 2012;17(4):385–90. Epub 2013/01/03. doi: 10.1111/j.1529-8027.2012.00436.x . 23279339

[11] Waddington-Cruz M, Schmidt H, Botteman MF, Carter JA, Stewart M, Hopps M, et al. Epidemiological and clinical characteristics of symptomatic hereditary transthyretin amyloid polyneuropathy: a global case series. Orphanet J Rare Dis. 2019;14(1):34. Epub 2019/02/10. doi: 10.1186/s13023-019-1000-1 ; PubMed Central PMCID: PMC6368811. 30736835 PMC6368811

[12] Mazzeo A, Russo M, Di Bella G, Minutoli F, Stancanelli C, Gentile L, et al. Transthyretin-related familial amyloid polyneuropathy (TTR-FAP): a single-center experience in Sicily, an Italian endemic area. J Neuromuscul Dis. 2015;2(s2):S39–S48. Epub 2015/07/22. doi: 10.3233/JND-150091 ; PubMed Central PMCID: PMC5271421. 27858761 PMC5271421

[13] Damy T, Kristen AV, Suhr OB, Maurer MS, Plante-Bordeneuve V, Yu CR, et al. Transthyretin cardiac amyloidosis in continental Western Europe: an insight through the Transthyretin Amyloidosis Outcomes Survey (THAOS). Eur Heart J. 2019;43(5):391–400. Epub 2019/04/03. doi: 10.1093/eurheartj/ehz173 ; PubMed Central PMCID: PMC8825236. 30938420 PMC8825236

[14] Gagliardi C, Perfetto F, Lorenzini M, Ferlini A, Salvi F, Milandri A, et al. Phenotypic profile of Ile68Leu transthyretin amyloidosis: an underdiagnosed cause of heart failure. Eur J Heart Fail. 2018;20(10):1417–25. Epub 2018/08/03. doi: 10.1002/ejhf.1285 . 30070416

[15] Silva Batista JAD, Carrera LR, Viriato ARF, Novaes MAC, de Morais RJL, Oliveira FTO, et al. Involvement of cranial nerves in ATTR Ile127Val amyloidosis. Eur J Med Genet. 2022;65(7):104524. Epub 2022/05/18. doi: 10.1016/j.ejmg.2022.104524 . 35580748

[16] Nanri K, Utsumi H, Yamada M, Takata Y, Matsumura A, Kougo K, et al. Transthyretin Val 107 in a Japanese patient with familial amyloid polyneuropathy. J Neurol Sci. 2002;198(1–2):93–6. Epub 2002/06/01. doi: 10.1016/s0022-510x(02)00051-5 . 12039669

[17] Cassereau J, Lavigne C, Letournel F, Ghali A, Verny C, Dubas F, et al. Hereditary amyloid neuropathy by transthyretin Val107 mutation in a patient of African origin. J Peripher Nerv Syst. 2008;13(3):251–4. Epub 2008/10/11. doi: 10.1111/j.1529-8027.2008.00185.x . 18844793

[18] Davion JB, Bocquillon P, Cassim F, Frezel N, Lacour A, Dhaenens CM, et al. Electro-clinical presentation of hereditary transthyretin related amyloidosis when presenting as a polyneuropathy of unknown origin in northern France. Rev Neurol (Paris). 2021;177(9):1160–7. Epub 2021/07/14. doi: 10.1016/j.neurol.2021.02.392 . 34253345

[19] Mariani LL, Lozeron P, Theaudin M, Mincheva Z, Signate A, Ducot B, et al. Genotype-phenotype correlation and course of transthyretin familial amyloid polyneuropathies in France. Ann Neurol. 2015;78(6):901–16. Epub 2015/09/16. doi: 10.1002/ana.24519 ; PubMed Central PMCID: PMC4738459. 26369527 PMC4738459

[20] Plante-Bordeneuve V, Suhr OB, Maurer MS, White B, Grogan DR, Coelho T. The Transthyretin Amyloidosis Outcomes Survey (THAOS) registry: design and methodology. Curr Med Res Opin. 2013;29(1):77–84. Epub 2012/12/01. doi: 10.1185/03007995.2012.754349 . 23193943

[21] Coelho T, Vinik A, Vinik EJ, Tripp T, Packman J, Grogan DR. Clinical measures in transthyretin familial amyloid polyneuropathy. Muscle Nerve. 2017;55(3):323–32. Epub 2016/07/17. doi: 10.1002/mus.25257 . 27422379

[22] Pickard AS, Law EH, Jiang R, Pullenayegum E, Shaw JW, Xie F, et al. United States valuation of EQ-5D-5L health states using an international protocol. Value Health. 2019;22(8):931–41. Epub 2019/08/21. doi: 10.1016/j.jval.2019.02.009 . 31426935

[23] Dispenzieri A, Coelho T, Conceicao I, Waddington-Cruz M, Wixner J, Kristen AV, et al. Clinical and genetic profile of patients enrolled in the Transthyretin Amyloidosis Outcomes Survey (THAOS): 14-year update. Orphanet J Rare Dis. 2022;17(1):236. Epub 2022/06/19. doi: 10.1186/s13023-022-02359-w ; PubMed Central PMCID: PMC9206752. 35717381 PMC9206752

[24] Leibou L, Frand J, Sadeh M, Lossos A, Kremer E, Livneh A, et al. Clinical and genetic findings in eight Israeli patients with transthyretin-associated familial amyloid polyneuropathy. Isr Med Assoc J. 2012;14(11):662–5. Epub 2012/12/18. . 23240369

[25] Rapezzi C, Riva L, Quarta CC, Perugini E, Salvi F, Longhi S, et al. Gender-related risk of myocardial involvement in systemic amyloidosis. Amyloid. 2008;15(1):40–8. Epub 2008/02/13. doi: 10.1080/13506120701815373 . 18266120

[26] Santos D, Santos MJ, Alves-Ferreira M, Coelho T, Sequeiros J, Alonso I, et al. mtDNA copy number associated with age of onset in familial amyloid polyneuropathy. J Neurol Neurosurg Psychiatry. 2018;89(3):300–4. Epub 2017/10/12. doi: 10.1136/jnnp-2017-316657 . 29018163

