# Supplementary material for: Phenotypic characteristics of F64L, I68L, I107V, and S77Y ATTRv genotypes from the Transthyretin Amyloidosis Outcomes Survey (THAOS)
Source: PLoS One. 2024 Jan 19;19(1):e0292435. doi: 10.1371/journal.pone.0292435 (PMC10798432; doi:10.1371/journal.pone.0292435)
Supplement: S1 Table — (DOCX) [file pone.0292435.s001.docx]

**S1 Table. Baseline demographic and clinical characteristics of symptomatic patients with ATTRv amyloidosis and the F64L variant in THAOS, detailed by country of origins.**

| **Characteristic** | **All countries**  **(N = 46)** | **Italy**  **(n = 29)** | **United States**  **(n = 15)** | **Argentina**  **(n = 1)** | **Brazil**  **(n = 1)** |
| --- | --- | --- | --- | --- | --- |
| Male, n (%) | 31 (67.4) | 16 (55.2) | 13 (86.7) | 1 (100) | 1 (100) |
| Age at enrollment, median (10th, 90th percentile), years | 67.6 (52.1, 76.4) | 67.5 (52.1, 76.4) | 66.8 (47.3, 76.5) | 76.2(76.2, 76.2) | 67.9 (67.9, 67.9) |
| BMI |  |  |  |  |  |
| Median (10th, 90th percentile) | 23.8 (19.1, 32.3) | 22.7 (18.8, 27.5) | 27.0 (21.2, 38.1) | 22.5 (22.5, 22.5) | 27.1 (27.1, 27.1) |
| mBMI | n = 22 | n = 13 | n = 9 | n = 0 | n = 0 |
| Median  (10th, 90^th^ percentile) | 1083.5  (805.2, 1389.1) | 1041.0 (646.5, 1269.5) | 1217.1 (843.1, 1868.0) | — |  |
| Duration of ATTRv amyloidosis symptoms  Median (10th, 90th percentile), years | 5.0  (0.8, 13.4) | 5.5 (1.7, 15.2) | 3.2 (0.3, 9.1) | 9.7 (9.7, 9.7) | 9.4 (9.4, 9.4) |
| EQ-5D-3L index score | n = 20 | n = 13 | n = 6 | n = 0 | n = 1 |
| Median (10th, 90th percentile) | 0.60 (0.31, 0.82) | 0.60 (0.31, 0.83) | 0.69 (0.44, 0.80) | - | 0.38 (0.38, 0.38) |
| Derived NIS-LL total score^a^ | n = 12 | n = 7 | n = 4 | n = 0 | n = 1 |
| Median  (10th, 90th percentile) | 23.6 (2.0, 61.0) | 31.3 (0.0, 67.0) | 12.0 (2.0, 40.0) | - | 61.0 (61.0, 61.0) |
| Karnofsky Performance Status score^b^, n (%) | n = 34 | n = 28 | n = 4 | n = 1 | n = 1 |
| 10–30 | 0 | 0 | 0 | 0 |  |
| 40–60 | 10 (29.4) | 8 (28.6) | 0 | 1 (100.0) | 1 (100.0) |
| 70–90 | 18 (53.0) | 14 (50.0) | 4 (100.0) | 0 |  |
| 100 | 6 (17.6) | 6 (21.4) | 0 | 0 |  |

^a^NIS-LL score was derived by an algorithm using symptoms.

^b^Percentages based on number of patients with available scores.

ATTRv amyloidosis, hereditary transthyretin amyloidosis; BMI, body mass index; mBMI, modified body mass index; NIS-LL, Neuropathy Impairment Score in the Lower Limbs; THAOS, Transthyretin Amyloidosis Outcomes Survey.
