# Supplementary material for: Phenotypic characteristics of F64L, I68L, I107V, and S77Y ATTRv genotypes from the Transthyretin Amyloidosis Outcomes Survey (THAOS)
Source: PLoS One. 2024 Jan 19;19(1):e0292435. doi: 10.1371/journal.pone.0292435 (PMC10798432; doi:10.1371/journal.pone.0292435)
Supplement: S2 Table — (DOCX) [file pone.0292435.s002.docx]

**S2 Table. Baseline demographic and clinical characteristics of symptomatic patients with ATTRv amyloidosis and the I68L variant in THAOS, detailed by country of origins.**

| **Characteristic** | **All countries**  **(N = 45)** | **Italy**  **(n = 39)** | **United States**  **(n = 4)** | **Germany**  **(n = 1)** | **France**  **(n = 1)** |
| --- | --- | --- | --- | --- | --- |
| Male, n (%) | 35 (77.8) | 31 (79.5) | 3 (75.0) | 1 (100) | 0 |
| Age at enrollment, median (10th, 90th percentile), years | 69.3 (56.5, 80.4) | 69.7 (54.8, 80.4) | 68.1 (59.2, 70.4) | 64.6 (64.6, 64.6) | 71.1 (71.1, 71.1) |
| Duration of ATTRv amyloidosis symptoms, median (10th, 90th percentile), years | 3.0 (0.7, 11.9) | 3.0 (0.7, 13.0) | 2.3 (0.5, 3.6) | 5.0 (5.0, 5.0) | 5.6 (5.6, 5.6) |
| BMI, median (10th, 90th percentile) | 25.6 (22.0, 32.4) | 25.5 (20.7, 29.4) | 29.9 (24.3, 34.7) | 34.6 (34.6, 34.6) | 24.6 (24.6, 24.6) |
| mBMI, n | 9 | 3 | 4 | 1 | 1 |
| Median (10th, 90th percentile) | 1106.4 (690.9, 1539.8) | 1106.4 (690.9, 1275.4) | 1128.1 (962.4, 1253.9) | 1539.8 (1540, 1539.8) | 830.4 (830.4, 830.4) |
| EQ-5D-3L index score, n | 39 | 36 | 3 | 0 | 0 |
| Median (10th, 90th percentile) | 0.8 (0.6, 1.0) | 0.8 (0.6, 1.0) | 0.8 (0.8, 1.0) |  |  |
| NIS-LL total, n | 4 | 3 | 0 | 1 | 0 |
| Median (10th, 90th percentile) | 2.0 (0.0, 20.0) | 2.0 (0.0, 20.0) |  | 2.0 (2.0, 2.0) |  |
| Karnofsky Performance Status score^a^, n (%) |  |  |  |  |  |
| 10–30 | 0 | 0 | 0 | 0 | 0 |
| 40–60 | 4 (9.7) | 3 (7.9) | 1 (100) | 0 | 0 |
| 70–90 | 28 (68.3) | 26 (68.4) | 0 | 1 (100) | 1 (100) |
| 100 | 9 (22.0) | 9 (23.7) | 0 | 0 | 0 |

^a^Percentages based on number of patients with available scores.

ATTRv amyloidosis, hereditary transthyretin amyloidosis; BMI, body mass index; mBMI, modified body mass index; NIS-LL, Neuropathy Impairment Score in the Lower Limbs; THAOS, Transthyretin Amyloidosis Outcomes Survey.
