# Supplementary material for: Phenotypic characteristics of F64L, I68L, I107V, and S77Y ATTRv genotypes from the Transthyretin Amyloidosis Outcomes Survey (THAOS)
Source: PLoS One. 2024 Jan 19;19(1):e0292435. doi: 10.1371/journal.pone.0292435 (PMC10798432; doi:10.1371/journal.pone.0292435)
Supplement: S3 Table — (DOCX) [file pone.0292435.s003.docx]

**S3 Table. Baseline demographic and clinical characteristics of symptomatic patients with ATTRv amyloidosis and the I107V variant in THAOS, detailed by country of origins.**

| **Characteristic** | **All countries**  **(N = 21)** | **France**  **(n = 8)** | **Brazil**  **(n = 6)** | **Germany**  **(n = 4)** | **Japan**  **(n = 2)** | **United States**  **(n = 1)** |
| --- | --- | --- | --- | --- | --- | --- |
| Male, n (%) | 18 (85.7) | 6 (75.0) | 6 (100) | 3 (75.0) | 2 (100) | 1 (100) |
| Age at enrollment, median (10th, 90th percentile), years | 63.7 (56.7, 76.0) | 63.6 (56.4, 76.0) | 62.6 (59.1, 78.3) | 67.1 (54.8, 77.8) | 67.7 (61.4, 74.1) | 69.6 (69.6, 69.6) |
| Duration of ATTRv amyloidosis symptoms, median (10th, 90th percentile), years | 3.6 (1.2, 10.6) | 3.5 (0.5, 16.3) | 3.0 (1.7, 14.9) | 5.6 (3.3, 10.3) | 5.9 (1.2, 10.6) | 5.7 (5.7, 5.7) |
| BMI, n | 18 | 8 | 4 | 3 | 2 | 1 |
| Median (10th, 90th percentile) | 25.9 (19.5, 32.6) | 25.8 (19.5, 32.6) | 27.9 (19.5, 37.8) | 25.8 (22.9, 30.8) | 24.8 (22.1, 27.6) | 23.8 (23.8, 23.8) |
| mBMI, n | 10 | 4 | 1 | 2 | 2 | 1 |
| Median (10th, 90th percentile) | 879.1 (824.9, 1446.2) | 922.0 (862.0, 1760.7) | 879.7 (879.7, 879.7) | 922.6 (878.5, 966.8) | 962.4 (793.1, 1131.8) | 856.7 (856.7, 856.7) |
| EQ-5D-5L index score, n | 12 | 3 | 5 | 3 | 0 | 1 |
| Median (10th, 90th percentile) | 0.6 (0.1, 0.9) | 0.8 (0.8, 1.0) | 0.5 (0.1, 0.6) | 0.7 (0.1, 0.9) |  | 0.8 (0.8, 0.8) |
| NIS-LL total, n | 10 | 2 | 6 | 2 | 0 | 0 |
| Median (10th, 90th percentile) | 38.9 (2.0, 65.9) | 2.0 (0.0, 4.0) | 57.5 (12.0, 70.5) | 33.5 (29.0, 38.0) |  |  |
| Karnofsky Performance Status score^a^, n (%) |  |  |  |  |  |  |
| 10–30 | 0 | 0 | 0 | 0 | 0 | 0 |
| 40–60 | 7 (41.2) | 2 (33.3) | 3 (50.0) | 2 (66.7) | 0 | 0 |
| 70–90 | 7 (41.2) | 1 (16.7) | 3 (50.0) | 1 (33.3) | 1 (33.3) | 1 (33.3) |
| 100 | 3 (17.6) | 3 (50.0) | 0 | 0 | 0 | 0 |

^a^Percentages based on number of patients with available scores.

ATTRv amyloidosis, hereditary transthyretin amyloidosis; BMI, body mass index; mBMI, modified body mass index; NIS-LL, Neuropathy Impairment Score in the Lower Limbs; THAOS, Transthyretin Amyloidosis Outcomes Survey.s
