# Supplementary material for: Phenotypic characteristics of F64L, I68L, I107V, and S77Y ATTRv genotypes from the Transthyretin Amyloidosis Outcomes Survey (THAOS)
Source: PLoS One. 2024 Jan 19;19(1):e0292435. doi: 10.1371/journal.pone.0292435 (PMC10798432; doi:10.1371/journal.pone.0292435)
Supplement: S4 Table — (DOCX) [file pone.0292435.s004.docx]

**S4 Table. Baseline demographic and clinical characteristics of symptomatic patients with ATTRv amyloidosis and the S77Y variant in THAOS, detailed by country of origins.**

| **Characteristic** | **All countries**  **(N = 29)** | **France**  **(n = 16)** | **Israel**  **(n = 5)** | **United States**  **(n = 5)** | **Spain**  **(n = 3)** |
| --- | --- | --- | --- | --- | --- |
| Male, n (%) | 21 (72.4) | 11 (68.8) | 4 (80.0) | 5 (100) | 1 (33.3) |
| Age at enrollment, median (10th, 90th percentile), years | 57.8 (47.2, 69.6) | 57.8 (45.0, 65.7) | 60.7 (55.0, 65.2) | 57.8 (50.0, 73.1) | 61.0 (53.9, 74.6) |
| Duration of ATTRv amyloidosis symptoms, median (10th, 90th percentile), years | 4.7 (1.2, 12.7) | 5.9 (1.3, 11.4) | 3.0 (0.7, 12.7) | 6.3 (1.2, 8.4) | 20.2 (4.0, 34.4) |
| BMI, n | 27 | 16 | 5 | 4 | 2 |
| Median (10th, 90th percentile) | 25.3 (19.9, 33.1) | 24.7 (19.9, 33.1) | 27.6 (16.6, 41.3) | 28.0 (24.5, 29.8) | 25.2 (23.4, 27.0) |
| mBMI, n | 10 | 5 | 2 | 1 | 2 |
| Median (10th, 90th percentile) | 978.0 (564.0, 1228.6) | 961.7 (530.0, 1216.8) | 796.1 (597.9, 994.3) | 891.7 (891.7, 891.7) | 1147.2 (1054, 1240.3) |
| EQ-5D-3L index score, n | 2 | 0 | 0 | 0 | 2 |
| Median (10th, 90th percentile) | 0.7 (0.7, 0.8) | - |  |  | 0.7 (0.7, 0.8) |
| NIS-LL total, n | 24 | 16 | 4 | 2 | 2 |
| Median (10th, 90th percentile) | 10.0 (0.0, 10.0) | 10.0 (0.0, 10.0) | 10.0 (6.0, 10.0) | 5.0 (0.0, 10.0) | 10.0 (10.0, 10.0) |
| Karnofsky Performance Status score^a^, n (%) |  |  |  |  |  |
| 10–30 | 1 (4.8) | 1 (6.2) | 0 | 0 | 0 |
| 40–60 | 4 (19.1) | 2 (12.5) | 2 (66.7) | 0 | 0 |
| 70–90 | 12 (57.1) | 10 (62.5) | 1 (33.3) | 0 | 1 (50.0) |
| 100 | 4 (19.0) | 3 (18.8) | 0 | 0 | 1 (50.0) |

^a^Percentages based on number of patients with available scores.

ATTRv amyloidosis, hereditary transthyretin amyloidosis; BMI, body mass index; mBMI, modified body mass index; NIS-LL, Neuropathy Impairment Score in the Lower Limbs; THAOS, Transthyretin Amyloidosis Outcomes Survey.
